# Heating and Ventilation

**Published:** 1905-07-15

**Authors:** W.F. Barrett

**Affiliations:** Royal College of Science, Dublin


### EDITOR'S LETTER-BOX.

[Our Correspondents are reminded that prolixity is a great bar to publication, and that brevity of style and conciseness of statement greatly facilitate early insertion.]

### HEATING AND VENTILATION.

SIR,—A contributor to your columns makes a violent attack upon a paper that I was urgently requested to contribute to the recent annual meeting of the Institute of Heating and Ventilating Engineers.

Nothing is more welcome than instructive criticism, but anything more grotesque than your correspondent's criticism it would be hard to conceive. For instance, I explained in my paper, a copy of which I enclose, how it was that a metal globe placed over a luminous gas flame warmed a room more efficiently than the naked flame; this paradoxical result being due, as I showed, to heating the room chiefly by radiation from the globe. Your correspondent says "the professor was unable to explain the result . . . It never appears to have occurred to him that the result was due to more perfect combustion." As a matter of fact the combustion is less perfect with the globe (which is closed above) than without it. Then your correspondent goes on to say I spoke of the deposition of dust on cool surfaces, but "beyond a list of names of eminent men who had conducted some experiments . . . he was unable to clear up the mystery." You will see by reference to my paper that the latter part of it was entirely devoted to explaining this curious phenomenon. Then he speaks of my knowing nothing of the Plenum system of ventilation, which I discussed at some length and gave its advantages and disadvantages.

I should not have troubled you with this letter if your readers had had the opportunity of reading my paper for themselves. I enclose the published comments of the experienced engineers who took part in the discussion.

Yours, obediently,

W. F. BARRETT.

Royal College of Science, Dublin.

